# Two-Stage Micturition as a Clue to Giant Inguinoscrotal Bladder Herniation: A Rare Diagnostic Pitfall

**DOI:** 10.7759/cureus.111220

**Published:** 2026-06-21

**Authors:** Yassine Ameur, Meriem Zhim, Abdelhakim Oukouhou, Alaa Sobhi, Larbi Hamedoun, Mohamed Tetou, Mohammed Mrabti, Youness Boukhlifi, Abdessamad El Bahri, Mohammed Alami, Ahmed Ameur

**Affiliations:** 1 Urology, Faculty of Medicine and Pharmacy of Rabat/Mohammed V Military Teaching Hospital, Rabat, MAR; 2 Radiology, Faculty of Medicine and Pharmacy of Rabat/Mohammed V Military Teaching Hospital, Rabat, MAR; 3 Urology, Mohammed V Military Teaching Hospital, Rabat, MAR

**Keywords:** bladder outlet obstruction, computed tomography, inguinoscrotal bladder hernia, lower urinary tract symptoms, mery's sign, nocturia, two-stage micturition

## Abstract

Inguinal bladder herniation is a rare condition and is frequently underdiagnosed because of its nonspecific presentation. We report the case of a 68-year-old man who presented with progressively worsening lower urinary tract symptoms, including frequency, nocturia, weak urinary stream, elevated post-void residual volume, and a slowly enlarging right inguinoscrotal swelling. The coexistence of severe voiding symptoms and obstructive urinary parameters initially suggested benign prostatic obstruction. However, the presence of two-stage micturition requiring manual compression of the scrotal mass to complete bladder emptying (Mery’s sign) raised suspicion of bladder involvement. Contrast-enhanced computed tomography with delayed excretory-phase acquisition revealed a giant right inguinoscrotal bladder hernia with marked distortion of the bladder contour, while three-dimensional reconstruction provided precise anatomical characterization and facilitated surgical planning. The patient underwent successful surgical repair with complete resolution of urinary symptoms. This case highlights the diagnostic challenge posed by giant inguinoscrotal bladder hernias, particularly when they mimic common causes of bladder outlet obstruction, and emphasizes the importance of recognizing Mery’s sign and performing preoperative cross-sectional imaging to establish the diagnosis, prevent iatrogenic bladder injury, and optimize surgical management.

## Introduction

Inguinal bladder herniation is a rare entity in daily urological practice, representing approximately 1-4% of inguinal hernias and up to 10% in elderly obese men [1,2]. Predisposing factors include advanced age, male sex, chronic bladder outlet obstruction, obesity, weakening of the abdominal wall, and prolonged increases in intra-abdominal pressure [3]. Although most bladder hernias are asymptomatic, larger hernias may present with lower urinary tract symptoms (LUTS), inguinal or suprapubic swelling, urinary retention, recurrent urinary tract infection, or obstructive uropathy [4].

The condition remains frequently overlooked preoperatively, with historical diagnostic rates below 15%, resulting in potential intraoperative bladder injury during hernia repair [5]. Cross-sectional imaging, particularly contrast-enhanced computed tomography (CT) during the excretory phase, has substantially improved diagnostic accuracy and anatomical characterization [6].

We report a rare case of suprapubic inguinal bladder hernia presenting with LUTS in an elderly patient, highlighting the importance of modern CT imaging and preoperative recognition.

## Case presentation

A 68-year-old man presented with a long-standing history of progressively worsening LUTS, including urinary frequency, nocturia, weak urinary stream, hesitancy, and a persistent sensation of incomplete bladder emptying. These symptoms had gradually evolved over several years and had become increasingly bothersome during the months preceding presentation. The patient also reported intermittent episodes of acute urinary retention that resolved spontaneously, as well as recurrent urinary tract infections treated empirically in the community. Concurrently, the patient had noticed a slowly enlarging right inguinoscrotal swelling, which had been present for several years but had markedly increased in size over the preceding months. The swelling was initially asymptomatic and reducible but progressively became irreducible and associated with a sensation of pelvic heaviness and discomfort during physical activity.

A particularly unusual feature of the patient’s history was the presence of two-stage micturition. Following apparent completion of voiding, he frequently experienced persistent suprapubic fullness and the sensation of residual urine. He had learned to manually compress the right inguinoscrotal mass to facilitate evacuation of an additional volume of urine, a finding highly suggestive of Mery’s sign and indicative of bladder involvement within the hernia sac. This symptom had been present for several months but had not been previously recognized as a manifestation of bladder herniation. Initially, the symptoms were attributed to benign prostatic obstruction, a far more common condition in elderly men. However, the persistence of voiding dysfunction despite medical management and the presence of two-stage urination raised suspicion of an underlying anatomical abnormality involving the lower urinary tract.

Physical examination revealed a large right inguinoscrotal swelling extending to the suprapubic region, partially reducible and increasing with the Valsalva maneuver. Digital rectal examination showed a moderately enlarged, smooth, non-tender prostate without suspicious nodules. The patient's International Prostate Symptom Score (IPSS) was 24/35, indicating severe LUTS, with a quality-of-life score of 5/6. Uroflowmetry demonstrated a maximum urinary flow rate (Qmax) of 7 mL/s, and post-void residual urine volume (PVR) measured by ultrasound was 180 mL.

Contrast-enhanced abdominopelvic CT with delayed excretory-phase imaging revealed a large right inguinoscrotal hernia containing a substantial portion of the urinary bladder. The herniated bladder component extended through the inguinal canal into the scrotum, resulting in marked distortion of the bladder contour and confirming continuity between the herniated segment and the native bladder (Figure 1).

**Figure 1:**
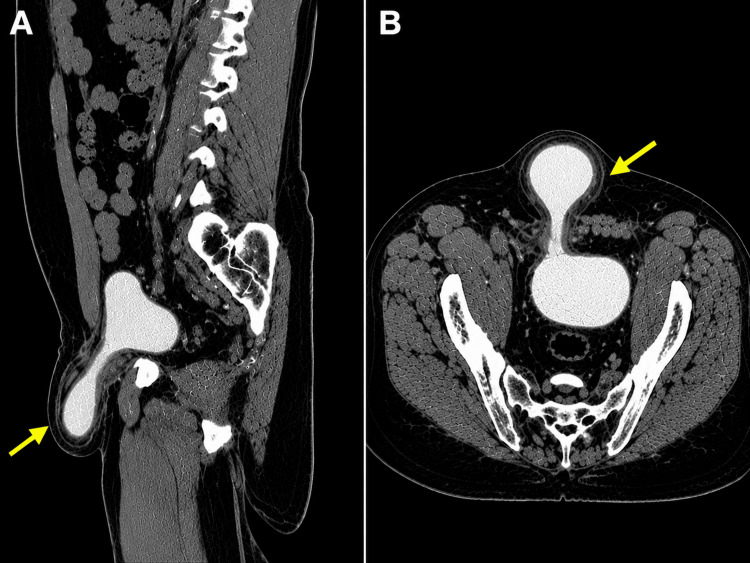
Contrast-enhanced CT images obtained during the delayed excretory phase demonstrating a giant right inguinoscrotal bladder hernia. (A) Sagittal reconstruction showing herniation of the bladder through the inguinal canal into the scrotum; (B) Axial image confirming continuity between the herniated bladder component and the native bladder. Yellow arrows highlight the herniated bladder component within the scrotum.

Three-dimensional (3D) volume-rendered reconstruction further demonstrated the extent of bladder displacement and provided a comprehensive visualization of the hernia morphology, highlighting the deformation of the opacified bladder and its relationship with the surrounding pelvic structures (Figure 2).

**Figure 2:**
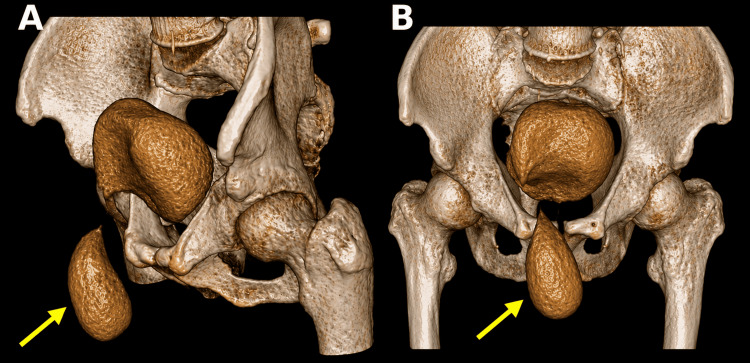
Three-dimensional volume-rendered CT reconstruction illustrating the morphology and extent of the giant inguinoscrotal bladder hernia. (A) Oblique lateral view demonstrating bladder displacement through the inguinal canal; (B) Frontal view highlighting deformation of the bladder contour and the relationship between the pelvic and herniated bladder components. Yellow arrows highlight the herniated bladder component within the scrotum.

Although precise volumetric analysis was not performed, imaging suggested that the herniated component accounted for a substantial proportion of the bladder volume, estimated at approximately 30-40%, highlighting the magnitude of bladder involvement. These imaging findings were essential for establishing the diagnosis and facilitating preoperative surgical planning.

Following the diagnosis, the patient underwent surgical repair with reduction of the herniated bladder and hernia repair. Postoperatively, the patient experienced complete resolution of the two-stage micturition and lower urinary tract symptoms, with no recurrence during follow-up.

## Discussion

Bladder herniation: a rare but probably underdiagnosed condition

Inguinal bladder herniation represents an uncommon form of sliding hernia and is estimated to account for 1-4% of all inguinal hernias. Although traditionally considered rare, recent evidence suggests that its incidence may be underestimated because many patients remain asymptomatic or are diagnosed incidentally during surgery. Elderly men constitute the most affected population, particularly those with chronic bladder outlet obstruction, obesity, decreased bladder tone, and weakened abdominal wall musculature. These factors collectively contribute to chronic elevation of intravesical pressure and progressive bladder displacement through the inguinal canal. Recent reports continue to emphasize that Inguinal Bladder Hernia remains a frequently overlooked diagnosis despite advances in cross-sectional imaging and increased awareness among surgeons and radiologists [5].

Clinical spectrum and diagnostic pitfalls

The clinical presentation of bladder herniation is highly variable and depends on the volume of the herniated bladder component. While small hernias may remain asymptomatic, larger lesions commonly present with LUTS, urinary retention, recurrent urinary tract infections, hematuria, or inguinal and suprapubic swelling. The association of LUTS with a groin mass should raise suspicion for bladder involvement. Nevertheless, symptoms are frequently attributed to benign prostatic enlargement, resulting in delayed diagnosis. A classical but inconsistently reported sign is two-stage micturition, in which patients achieve complete bladder emptying only after manual compression of the hernia. Recent case reports indicate that many patients continue to be diagnosed intraoperatively, demonstrating that bladder herniation remains a significant diagnostic pitfall [7].

Understanding the diagnostic trap: bladder outlet obstruction or bladder herniation?

Elevated IPSS scores, impaired uroflowmetry parameters, and increased post-void residual volumes may misleadingly suggest benign bladder outlet obstruction, thereby delaying recognition of underlying bladder herniation. Because benign prostatic hyperplasia is highly prevalent in elderly men, LUTS is often attributed to prostatic obstruction. However, the coexistence of an inguinoscrotal mass, two-stage micturition, or the need for manual compression of the swelling to complete voiding should prompt consideration of bladder herniation. In such cases, cross-sectional imaging, particularly contrast-enhanced CT with delayed excretory-phase acquisition, is essential for establishing the diagnosis and guiding appropriate surgical management [8].

The diagnostic value of Mery’s sign

Mery’s sign, defined by a two-stage micturition pattern in which bladder emptying is completed after manual compression of an inguinoscrotal swelling, remains one of the most characteristic clinical findings of inguinal bladder herniation. Although rarely encountered in routine practice due to the uncommon nature of this condition, its presence should immediately raise suspicion of bladder involvement within the hernia sac. The sign reflects urine retention within the herniated portion of the bladder and is particularly observed in large inguinoscrotal hernias. Because LUTS frequently overlap with symptoms of benign prostatic obstruction, recognition of Mery’s sign may help avoid diagnostic delay and prompt appropriate imaging evaluation. Recent studies have highlighted that double voiding and scrotal compression-assisted bladder emptying remain highly suggestive clinical indicators of bladder herniation and may facilitate preoperative diagnosis, thereby reducing the risk of inadvertent bladder injury during surgical repair [9].

Role of contemporary imaging

Modern multidetector CT (MDCT) has transformed the diagnostic approach to bladder herniation. Historically, fewer than 15% of cases were identified preoperatively, whereas CT now allows reliable diagnosis before surgical intervention. Delayed excretory-phase imaging is particularly useful because it demonstrates contrast opacification within the herniated bladder segment and confirms its continuity with the native bladder. Furthermore, multiplanar and 3D reconstructions provide excellent anatomical characterization and facilitate surgical planning. Recent studies have highlighted that CT not only establishes the diagnosis but also identifies associated complications such as ureteral entrapment, hydronephrosis, bladder calculi, ischemic changes, and malignancy arising within the herniated component. Consequently, CT has become the imaging modality of choice for evaluating atypical inguinal hernias with urinary symptoms [2].

Diagnostic implications for surgical planning

The major clinical importance of preoperative recognition lies in preventing iatrogenic bladder injury. Unrecognized bladder involvement may expose patients to bladder perforation, urinary leakage, fistula formation, prolonged catheterization, infectious complications, and reoperation. Several contemporary reports have reiterated that inadvertent bladder injury remains one of the most important causes of morbidity associated with inguinal bladder hernia. Accurate preoperative imaging, therefore, has a direct impact on surgical safety and postoperative outcomes. Moreover, early diagnosis allows surgeons to anticipate anatomical complexity and select the most appropriate operative strategy, thereby reducing operative time and avoiding unnecessary bladder resection [10].

Surgical management in the modern era

Surgical repair remains the standard treatment for symptomatic bladder hernias. The preferred approach consists of careful reduction of the herniated bladder followed by tension-free mesh hernioplasty. Bladder resection is rarely required and should be reserved for specific situations such as necrosis, ischemia, tumors, diverticula, or irreducible nonviable bladder segments. Although open repair remains the most reported technique, laparoscopic and robotic-assisted approaches have gained increasing popularity. Recent publications have demonstrated that minimally invasive surgery offers excellent visualization of pelvic anatomy, facilitates bladder dissection, and may reduce postoperative pain and length of hospital stay in selected patients. In the present case, preoperative CT enabled accurate surgical planning, allowing uncomplicated reduction of the bladder and successful mesh repair without the need for partial cystectomy [11].

Clinical significance of the present case

Several aspects make this case noteworthy. First, the presence of suprapubic extension represents an unusual anatomical presentation that may further complicate diagnosis. Second, the coexistence of progressive LUTS and a suprapubic swelling illustrates a classic but frequently overlooked clinical scenario. Finally, the diagnosis was established preoperatively using delayed excretory-phase CT, highlighting the value of modern imaging in avoiding intraoperative surprises and preventing surgical complications.

The case was considered particularly challenging because of the rarity of massive bladder herniation, its insidious clinical presentation mimicking benign prostatic obstruction, and the significant risk of intraoperative bladder injury if the diagnosis had not been established preoperatively. The unusual combination of chronic LUTS, two-stage micturition, and giant inguinoscrotal bladder herniation underscored the diagnostic complexity of this condition and emphasized the pivotal role of cross-sectional imaging in surgical planning. This case therefore reinforces the importance of considering bladder herniation in elderly men presenting with LUTS associated with inguinal or suprapubic masses and supports systematic preoperative imaging in atypical hernia presentations.

## Conclusions

This case highlights a rare and clinically significant presentation of a giant inguinoscrotal bladder hernia masquerading as benign lower urinary tract dysfunction. The predominance of nonspecific urinary symptoms initially obscured the diagnosis, emphasizing the potential for bladder herniation to mimic more common urological conditions. In this context, the presence of two-stage micturition and Mery’s sign constituted important clinical clues that prompted further evaluation and ultimately led to the correct diagnosis. Awareness of these characteristic findings is essential to avoid diagnostic delays and inappropriate management in elderly men presenting with refractory lower urinary tract symptoms and associated inguinoscrotal swelling.

Beyond its unusual clinical presentation, this case underscores the considerable value of 3D CT reconstruction in the assessment of complex bladder hernias. The 3D images provided a comprehensive and intuitive depiction of the herniated bladder, clearly illustrating its volume, anatomical relationships, and extent of scrotal involvement. This enhanced visualization facilitated preoperative planning and contributed to safer surgical management by reducing the risk of inadvertent bladder injury. Given its rarity, striking radiological features, and educational relevance, this case demonstrates how advanced imaging can transform a complex anatomical abnormality into a practical surgical roadmap for both urologists and general surgeons.

## References

[1] Levine B (1951). Scrotal cystocele. J Am Med Assoc.

[2] Bacigalupo LE, Bertolotto M, Barbiera F, Pavlica P, Lagalla R, Mucelli RS, Derchi LE (2005). Imaging of urinary bladder hernias. AJR Am J Roentgenol.

[3] Gomella LG, Spires SM, Burton JM, Ram MD, Flanigan RC (1985). The surgical implications of herniation of the urinary bladder. Arch Surg.

[4] Bisharat M, O'Donnell ME, Thompson T, MacKenzie N, Kirkpatrick D, Spence RA, Lee J (2009). Complications of inguinoscrotal bladder hernias: a case series. Hernia.

[5] Kraft KH, Sweeney S, Fink AS, Ritenour CW, Issa MM (2008). Inguinoscrotal bladder hernias: report of a series and review of the literature. Can Urol Assoc J.

[6] Yustira RS, Haruman SP, Prasetyo RB, Triaswhoro CG (2025). Missed inguinoscrotal bladder hernia following herniorrhaphy: Diagnostic pitfalls and lessons from a rare case. Urol Case Rep.

[7] Davis DA, Glinski RW, Jones MC (2024). A rare case of complete inguinoscrotal bladder herniation with ureteric involvement: assessing diagnostic challenges and complex surgical management. Cureus.

[8] Alrabadi A, Al Demour S, Mansi H, AlHamss S, Al Omari L (2020). Evaluation of voiding position on uroflowmetry parameters and post void residual urine in patients with benign prostatic hyperplasia and healthy men. Am J Mens Health.

[9] Li M, Gu X (2026). Diagnostic challenges and surgical management of inguinal bladder hernia: a case report and literature review. Int J Abdom Wall Hernia Surg.

[10] Munasinghe BM, Jayasuriya NJ, Pathirana WP, Paranamanna RV, Jayalath MK, Karunarathna MW (2025). Management of a patient with an obstructed inguinal hernia with Amyand's variety: a report of a rare case with a review of the literature. Cureus.

[11] Mohammadi A, Golmohammadi P, Pakdel A, Aghamir SM (2025). Laparoscopic management of a recurrent, inguinal hernia containing urinary bladder: a case report and literature review. Case Rep Med.

